# Which severe COVID-19 patients could benefit from high dose dexamethasone? A Bayesian post-hoc reanalysis of the COVIDICUS randomized clinical trial

**DOI:** 10.1186/s13613-023-01168-z

**Published:** 2023-08-27

**Authors:** Sylvie Chevret, Lila Bouadma, Claire Dupuis, Charles Burdet, Jean-François Timsit, the COVIDICUS RCT group, Naima Beldjoudi, Sylvie Chevret, Charles Burdet, Céline Alloux, Fadila Amerali, Béatrice Andriss, Kamyl Baghli, Hélène Brocvielle, Florence Capelle, Ines Chibane, Sarra Dalibey, Nadia Ettalhaoui, Sabrine Lamri, Yohan Maurer, Jean-Marc Mintsa, Alice Oubahim, Marie-Capucine Tellier, Imane Zemihi, Lila Bouadma, Moustafa Abdel-Nabey, Billal Azzouguen, Ghenima Belkessa, Etienne De Montmollin, Veronique Deiler, Claire Dupuis, Aline Fallet, Guillaume Franchineau, Tiphaine Girard, Alexandra Grinea, Pierre Jaquet, Laura Kramer, Fariza Lamara, Lucie Lefevre, Mehdi Marzouk, Juliette Patrier, Simona Presente, Faiza Sayagh, Fabrice Sinnah, Romain Sonneville, Paul-Henri Wicky, Sylia Zmihi, Yves Cohen, Nathalie Barget, Rawan Belmokhtar, Sabrina Brahmi, Naoual Djabra, Nathan Ebstein, Souha Fliss, Amina Gourbdji, William Juguet, Fadhila Messani, Thomas Rambaud, Vanessa Rathouin, Mani Rebai, Marthe Rigal, Julien Schmidt, Marie Soulie, Yacine Tandjaoui-Lombiotte, Anaïs Winchenne, Marianne Ziol, Romain Arrestier, François Bagate, Morgan Benais, Ines Bendib, LE Lan, Brice Benelli, Enora Berti, Astrid Bertier, Guillaume Carteaux, Muriel Carvalho, Pedro Cavaleiro, Nicolas Deprost, Otto Hartman, Anne-Fleur Haudebourg, Delphine Lefebvre De Nailly, Julien Lopinto, Sabrina Mahiou, Paul Masi, Gaël Michaud, Luiza Nait-Chabane, Fariza Ouali, François Perier, Keyvan Razazi, Thiziri Sadaoui, Alaki Thiemele, Samuel Tuffet, Flavien Autron, Pauline Boddaert, Sylvie Brice, Morgan Caplan, Amélie Cerf, Nicolas Cousin, Marie Cuvelliez, Claire Delcourte, Arthur Durand, Raphaël Favory, Ahmed El Kalioubie, Myrtille Gaudel, Alexandre Gaudet, Julien Goutay, Marion Houard, Emmanuelle Jaillette, Mercé Jourdain, Geoffrey Ledoux, Laure Mariller, Guillaume Millot, Anne-Sophie Moreau, Christopher Niles, Saad Nseir, Thierry Onimus, Sébastien Preau, Aurélie Roucou, Anahita Rouze, Ouriel Saura, Arthur Simonnet, Romain Tortuyaux, Hamid Merdji, Hayat Allam, Jessy Cattelan, Raphaël Clere-Jehl, Julie Helms, Kévin Hilt, Anne Hutt-Clauss, Christine Kummerlen, Ferhat Meziani, Alexandra Monnier, Hassène Rahmani, Antoine Studer, Leonie Thiebaut, Aurélie Han Hew Wai, Charles Cerf, David Cortier, Jérôme Devaquet, Dimitri Fremont, Richard Galliot, Fabienne Juster, Mathilde Le-Marchand, Lucie Le-Meur, Mathilde Neuville, Emmanuel Roux, Guillaume Tachon, Camille Vassord-Dang, Benjamin Zuber, Cédric Bruel, Marie-José Aroulanda, Bryan Berthet-Delteil, Juliette Courtiade-Malher, Alix De Chevigny, Candice Fontaine, Julien Fournier, Sonia Garrigou, Meryam Jardin-Szucs, François Philippart, Sophie Renet, Emmanuelle Sacco, Marc Tran, Christophe Guitton, Delphine Bolle, Jean-Christophe Callahan, Nicolas Chudeau, Cédric Darreau, Séverine Guillarme, Mickael Landais, Laurent Latrouite, Charlène Le Moal, Eliott Lebasnier, Marie-Hélène Leroyer, Rémy Marnai, Juliette Meunier, Catherine Naveau, Marjorie Saint-Martin, Alain Robert, Patrice Tirot, Carole Schwebel, Joanna Bougnaud, Clara Candille, Roselyne Collomb-Muret, Charlotte Cordier, Louis-Marie Galerneau, Côme Gerard, Pierre Nicolas, Amel Refes, Guillaume Rigault, Florian Sigaud, Nicolas Terzi, Ester Terzi, Emmanuelle Turbil, Yann Vallod, Claire Dupuis, Mireille Adda, Claire Bachelier, Marine Bereiziat, Lise Bernard, Radhia Bouzgarrou, Laure Calvet, Pierre Couhault, Elisabeth Coupez, Frédéric Duee, Armelle Gilard, Tiphaine Girard, Kévin Grapin, Francis Kinda, Guillaume Laurichesse, Jean-Mathias Liteaudon, Bertrand Souweine, François Thouy, Mehdi Marzouk, Hervé Declercq, Dominique Descamps, Anne Dewatine, Sabine Janowski, Catherine Senis, Christophe Vinsonneau, Eric Kipnis, Anne Bignon, Tchadie Bommenel, Sylvie Brice, Claude Huriez, Matthieu Duprey, Pierre Garcon, Afef Hammami, Andréa Issad, Marie-Odile Jaccod-Deneuville, Safaâ Kachmar, Ly Van-Vong, Jonathan Zarka, Bruno Megarbane, Nicolas Deye, Aude Jacob, Isabelle Malissin, Aymen M’Rad, Sebastian Voicu, Guillaume Geri, Hélène Chambrin-Lauvray, Ouarda Douache, Mathieu Godement, Romain Jouffroy, Edouard Jullien, Matthieu Petit, Antoine Vieillard-Baron, Alain Cariou, Alexandre Boyer, Patricia Pavese, Bruno Giraudeau

**Affiliations:** 1 ECSTRRA, UMR 1153, Saint Louis Hospital, University Paris Cité, Paris, France; 2 Medical and Infectious Diseases ICU, APHP Bichat Hospital, 75018 Paris, France; 3 Université Paris Cité, IAME, INSERM, UMR 1137, 75018 Paris, France; 4 Intensive Care Unit, Gabriel Montpied Hospital, CHU de Clermont-Ferrand, 63000 Clermont-Ferrand, France; 5 Epidemiology, Biostatistics and Clinical Research Department, AP-HP, Bichat Hospital, 75018 Paris, France

**Keywords:** SARS-Cov-2, Steroids, Dose, Sepsis, Acute respiratory failure, Remdesivir, Critical care, ARDS, Bayesian analysis, Interaction

## Abstract

**Background:**

The respective benefits of high and low doses of dexamethasone (DXM) in patients with severe acute respiratory syndrome coronavirus 2 (SARS-Cov2) and acute respiratory failure (ARF) are controversial, with two large triple-blind RCTs reaching very important difference in the effect-size. In the COVIDICUS trial, no evidence of additional benefit of high-dose dexamethasone (DXM20) was found. We aimed to explore whether some specific patient phenotypes could benefit from DXM20 compared to the standard of care 6 mg dose of DXM (DXMSoC).

**Methods:**

We performed a post hoc exploratory Bayesian analysis of 473 patients who received either DXMSoc or DXM20 in the COVIDICUS trial. The outcome was the 60 day mortality rate of DXM20 over DXMSoC, with treatment effect measured on the hazard ratio (HR) estimated from Cox model. Bayesian analyses allowed to compute the posterior probability of a more than trivial benefit (HR < 0.95), and that of a potential harm (HR > 1.05). Bayesian measures of interaction then quantified the probability of interaction (Pr Interact) that the HR of death differed across the subsets by 20%. Primary analyses used noninformative priors, centred on HR = 1.00. Sensitivity analyses used sceptical and enthusiastic priors, based on null (HR = 1.00) or benefit (HR = 0.95) effects.

**Results:**

Overall, the posterior probability of a more than trivial benefit and potential harm was 29.0 and 51.1%, respectively. There was some evidence of treatment by subset interaction (i) according to age (Pr Interact, 84%), with a 86.5% probability of benefit in patients aged below 70 compared to 22% in those aged above 70; (ii) according to the time since symptoms onset (Pr Interact, 99%), with a 99.9% probability of a more than trivial benefit when lower than 7 days compared to a < 0.1% probability when delayed by 7 days or more; and (iii) according to use of remdesivir (Pr Interact, 91%), with a 90.1% probability of benefit in patients receiving remdesivir compared to 19.1% in those who did not.

**Conclusions:**

In this exploratory post hoc Bayesian analysis, compared with standard-of-care DXM, high-dose DXM may benefit patients aged less than 70 years with severe ARF that occurred less than 7 days after symptoms onset. The use of remdesivir may also favour the benefit of DXM20. Further analysis is needed to confirm these findings.

*Trial registration*: NCT04344730, date of registration April 14, 2020 (https://clinicaltrials.gov/ct2/show/NCT04344730?term=NCT04344730&draw=2&rank=1); EudraCT: 2020-001457-43 (https://www.clinicaltrialsregister.eu/ctr-search/search?query=2020-001457-43).

**Supplementary Information:**

The online version contains supplementary material available at 10.1186/s13613-023-01168-z.

## Background

In the early phases of the severe acute respiratory syndrome coronavirus 2 (SARS-CoV-2) pandemic, the benefit of low/intermediate doses of corticosteroids in patients with acute respiratory failure (ARF) was found by the RECOVERY trial [1], confirmed by a meta-analysis of ongoing studies [2]. Since then, dexamethasone (DXM) 6 mg has been included as a standard of care (SoC) in the management of SARS-CoV-2 ARF with oxygen requirements. However, despite 4 randomized clinical trials (RCTs) [3–6], including 2 triple-blind RCTs [3, 6], the benefit of high doses of corticosteroids (DXM 12 mg or more) compared to standard of care low/intermediate doses (DXMSoc) could not be demonstrated [7].

In the COVIDICUS multicentre randomized clinical trial [3], a total of 546 patients admitted to the intensive care unit (ICU) with SARS-CoV-2 acute respiratory hypoxemic failure (ARHF) were randomized 1:1 to either high-dose dexamethasone (DXM20, *n* = 270) or DXMSoC (*n* = 276) (NCT04344730). Such a strategy failed to have any impact on 60 day mortality (DXM20, 25.9%, vs. DXMSoC, 26.8%) [3]. However, the absence of any treatment effect, on the whole, may represent a benefit in some patients and harm in others due to differential treatment effects on subpopulations [8]. This was recently pointed out in the critical care setting, possibly related to the inclusion in trials of too heterogeneous populations [9]. Thus, exploring the treatment effect across different subgroups within an overall nonsignificant trial could be of interest [10].

Evaluation of the heterogeneity of the treatment effect is an essential aspect of personalized medicine and patient-centred outcome research. Factors that allow us to identify individuals who are more likely than others to experience a favourable or unfavourable effect of treatment define “predictive” factors, different from “prognostic” factors, defined as those used to identify the likelihood of a clinical event such as progression or death in patients. In patients with severe COVID-19 admitted to the ICU, several subsets of interest have been reported in the literature suggesting a prognostic [11–15] or predictive impact of those subsets [1, 5, 16–19].

However, to determine whether a factor is potentially predictive, a formal assessment of an interaction between the factor and treatment group needs to be performed [20]. Indeed, as with overall clinical trial results, chance findings are possible when assessing subgroup results. To assess the existence of interactions, the traditional approach evaluates the data in each of the subgroups independently and then uses several statistical tests for interaction, such as that of Gail and Simon [21]. However, clinical trials are rarely powered to detect statistically significant interactions. Bayesian approaches [22] have been reported as a novel solution to identify subgroups towards the “personalized medicine” [23]. Rather than postulating hypotheses regarding the quantity of interest, their main advantage is transparently communicating information by giving direct probabilistic statements [24]. In the setting of multi-population trials, Millen et al. [25] have proposed Bayesian interaction measures, referring to a potential concern that the inferences in the overall population may be unduly influenced by the treatment effect in a subgroup of patients.

In this study, based on the COVIDICUS trial, we used a Bayesian framework to assess the predictive value of several subsets of interest on the benefit of high-dose DXM in SARS-CoV-2 ARHF patients admitted to ICUs.

## Methods

### The covidicus trial

Study participation in the COVIDICUS trial (NCT04344730), sponsored by Assistance Publique-Hôpitaux de Paris (Paris, France), was proposed to all consecutive COVID-19 adult patients admitted to participating French ICUs who met the eligibility criteria. Eligible patients were adults aged ≥ 18 years admitted to the ICU within the last 48 h for confirmed or highly suspected COVID-19 infection and with signs of AHRF (PaO2 < 70 mmHg or transcutaneous oxygen saturation (SpO2) < 90% on room air, tachypnea > 30/min, laboured breathing, respiratory distress, or need for oxygen flow ≥ 6 L/min) and who could receive any available treatment intended to treat SARS-CoV-2 infection.

It aimed to compare the benefit of high-dose dexamethasone (DXM20, 20 mg/d for 5 days, then 10 mg/d × 5 days) compared to the standard of care (DXMSoC), first based on placebo. On July 3, 2020, after the publication of the recovery trial [1], the COVIDICUS Scientific Committee prompted the study group to amend the study protocol to allow investigators to administer DXM up to 6 mg/d for 10 days to DXMSoC patients. It also addressed the question of oxygen support technique, further comparing continuous positive airway pressure (CPAP) or high-flow oxygen therapy (HFNO) vs. standard oxygen support in non-intubated patients; however, we only focused on the effect of dexamethasone in this study.

Inclusions ranged from April 2020 to January 2021 in 19 French ICUs. The primary outcome was the time-to-all causes of death at Day 60 in the intent-to-treat population.

The trial was conducted in accordance with the Declaration of Helsinki. Signed informed consent was obtained from all included patients. An emergency consent procedure with the patient’s legal guardian or relatives was implemented for patients unable to consent.

### Patients

Of the 546 randomized patients, 73 were included before September 17, 2020, when the protocol was amended to switch the placebo control group to a low dose of dexamethasone. We included all 473 patients from the modified intention-to-treat (ITT) population who were enrolled thereafter and randomly allocated to either DXM high dose (DXM20, *n* = 234) or low dose (DXMSoC, *n* = 239).

### Subsets of interest

We first considered four partitions of patients based on (i) age (< , > 70 years), (ii) inflammatory status, defined at admission by either ferritin > 1000 μg/L or CRP > 100 mg/L, as previously reported [26]; (iii) time elapsed since the onset of COVID19 symptoms at admission, using 7 days as the threshold [1, 16, 27]; and (iv) fever (body temperature < 38 °C vs. $\ge$ 38 °C). Then, we also considered the effect according to values of CRP, ferritin and Ddimers, using the median value in the whole sample as the cut-off value (i.e., 135, 1120 and 940, respectively); for ferritin, we also considered the reported cut-off of 3150.29 μg/L, as used in [28]. We also considered the severity of the disease, as measured by the need of invasive mechanical ventilation (IMV) at study entry, or according to the median value of the SAPS2. Finally, we also tested the impact of the concomitant use of remdesivir.

### Statistical analysis

The treatment effect was defined as the hazard ratio (HR) of 60 day mortality in randomized groups DXM20 (high dose) vs. DXMSoC (low/moderate dose). We used the following Cox proportional hazards model $\lambda \left(t\right)={\lambda }_{0}\left(t\right)exp\left(\alpha t+\beta x+\gamma tx\right)$, where ${\lambda }_{0}\left(t\right)$ represents the baseline hazard function, $x$ denotes a binary covariate, $\beta$ the regression coefficient corresponding to the covariate, $t$ is a binary treatment indicator, $\alpha$ represents the treatment effect for patients with $x=0$, and $\gamma$ is the regression coefficient corresponding to the treatment-by-covariate interaction; treatment effect for patients with $x=1$ is thus given by $\alpha +\beta +\gamma$. The estimation of the regression coefficients in the Cox model was performed in a Bayesian framework, with baseline hazard function defined as a mixture of piecewise constant functions [29]. We considered a total number of knots *K* = 3 and an equally spaced partition of the time axis from 0 to 60 which corresponds to the longest survival time observed. The posterior distribution of each parameter was obtained, with the derived posterior density of any linear combination of the parameters, quantifying the uncertainty of treatment effect in each subset [30]. As proposed by Harrell in COVID-19 trials [31], a more than trivial benefit or a more than trivial harm was measured using the cut-off threshold of 1.05 on the HR scale. Thus, the posterior probability of a more than trivial benefit (HR < 0.95) and a more than trivial harm (HR > 1.05) overall and in each subset given the available data was computed. Then, according to Millen, treatment-by-subset interaction was measured on the ratio of treatment effect in the subsets, with the computed posterior probability that the HR of death in subsets differs by at least 20% [25].

Prior scenario was set under a non-informative independent framework with a gaussian N (0, 0.001) for each regression coefficient and an independent gamma distributions, Ga (0.01, 0.01) for each piecewise baseline hazard. Sensitivity analyses used optimistic and sceptical priors, that is centred on a positive (HR = 0.95) or null (HR = 1.00) effects, as recommended [32]. We also used Bayesian beta-binomial models, with the prevalence of death within the first 60 days following randomisation as the parameter of interest and the relative risk (RR) of death as the measure of effect.

We used R (https://www.R-project.org/) and JAGS [33], a user-friendly, open-source, validated software suited for the application of Bayesian methods, for analysis. We ran each model for 1000 burn-in simulations, then the model was run for 50,000 additional simulations to keep one in 10 so that a proper thinning is done. Gelman and Rubin’s convergence diagnostic [34] was computed. Trace plots of the sampled values for each parameter in the chain appear overlapping one another and Gelman–Rubin values were very close to 1, which indicated that convergence has been achieved.

## Results

The distribution of the 473 enrolled patients across the different strata is reported in Table 1. Most of the time, two subsets of imbalanced sizes were distinguished, with the lowest subset representing 11–29% of the sample.

**Table 1:** Characteristics of patients across treatment groups and baseline subsets

Baseline subsets at randomization	DXMSoC, *n* = 239 *N* (%)	DXM20, *n* = 234 *N* (%)
Age, years		
< 70	144 (60.2)	135 (57.7)
$\ge$ 70	95 (39.8)	99 (42.3)
Time since symptoms onset, days		
< 7	55 (23.1)	52 (22.9)
$\ge$ 7	183 (76.9)	175 (77.1)
Body Temperature, °C		
< 38	186 (79.1)	182 (78.1)
$\ge$ 38	49 (20.8)	51 (21.9)
Inflammation syndrome^a^		
No	26 (13.8)	23 (13.2)
Yes	162 (86.2)	151 (86.8)
Missing	51	60
Age < 60 and no inflammation*	3 (1.6)	10 (5.7)
Age $\ge$ 60 and inflammation	114 (60.6)	122 (70.1)
Age < 60 and inflammation	48 (25.5)	29 (16.7)
Age $\ge$ 60 and no inflammation	23 (12.2)	13 (7.5)
Missing	51	60
CRP, mg/L		
< 135	98 (49.7)	98 (50.0)
$\ge$ 135	99 (50.3)	98 (50.0)
Missing	42	38
Ferritin, μg/L		
< 1120	76 (55.5)	61 (44.2)
$\ge$ 1120	61 (44.5)	77 (55.8)
Missing	102	96
Ferritin, μg/L		
< 3150.29	123 (89.8)	121 (87.7)
$\ge$ 3150.29	14 (10.2)	17 (12.3)
Missing	102	96
D-Dimers, ng/mL		
< 940	89 (46.4)	103 (53.9)
$\ge$ 940	103 (53.6)	88 (46.1)
Missing	47	43
Mode of oxygenation		
O2 face mask	48 (20.1)	46 (19.7)
HFNO	48 (20.1)	41 (17.5)
CPAP	47 (19.7)	50 (21.4)
IMV	96 (40.1)	97 (41.4)
Invasive mechanical ventilation		
No	143 (59.9)	137 (58.6)
Yes	96 (40.1)	97 (41.4)
SAPS II		
< 33	113 (50.9)	100 (47.6)
$\ge$ 33	109 (49.1)	110 (52.4)
Other treatment of SARS-CoV-2		
Remdesivir	61 (25.5)	62 (26.5)
IL6	1 (0.4)	4 (1.7)

*CRP* C reactive protein, *O2* oxygen, *HFNO* High-Flow Nasal Oxygen, *CPAP* Continuous Positive Airway Pressure, *IMV* invasive mechanical ventilation, *IL6* interleukin 6, *SAPS II* Simplified Acute Physiology Score 2

^a^Defined as either ferritin level > 1000 μg/L or CRP > 100 mg/L

**Fig. 1:**
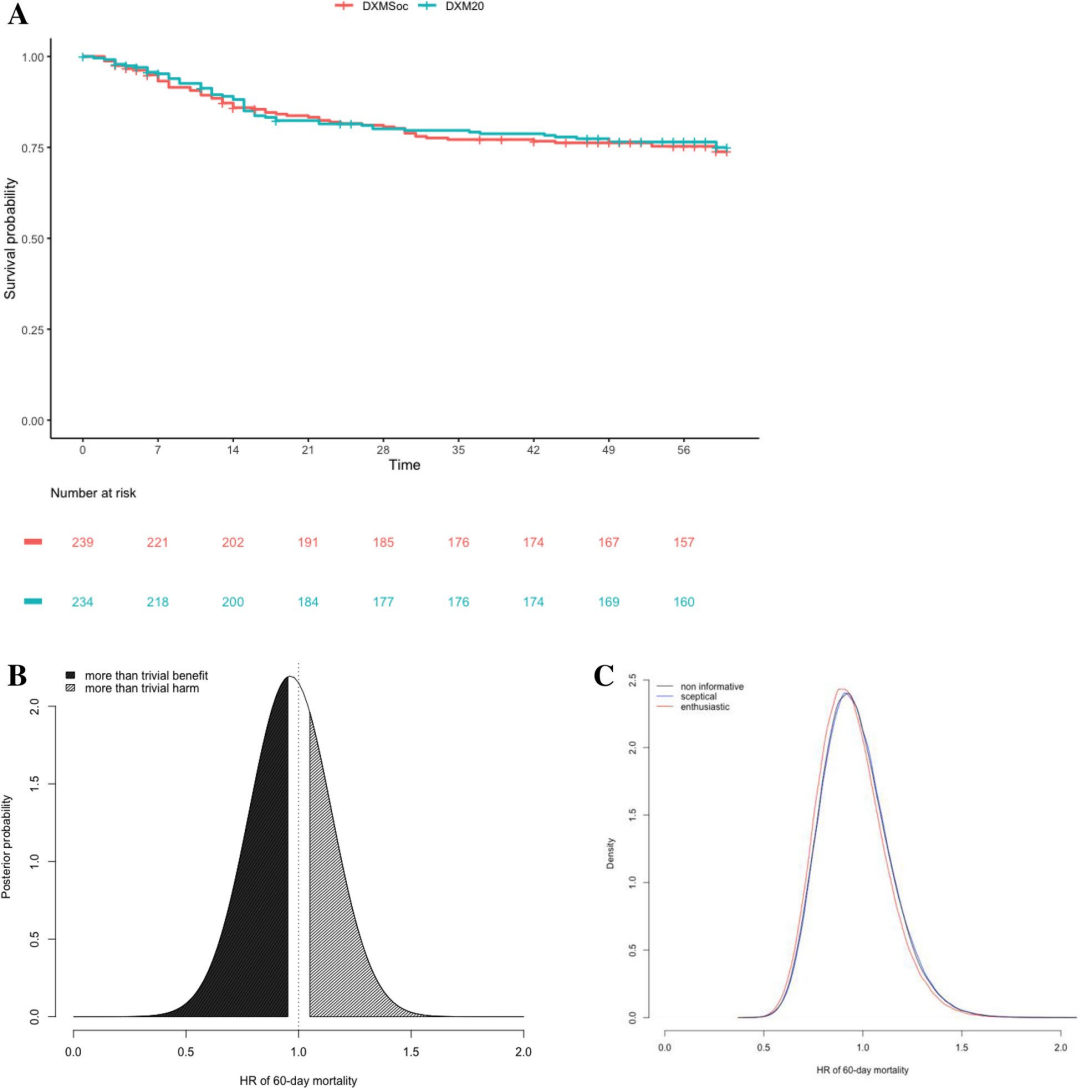
COVIDICUS Trial: Main trial outcome across the DXM randomized groups. Overall survival according to randomization (**a**) and posterior density of the hazard ratio (HR) of the 60-day mortality rate in the whole trial population based on a noninformative prior (**b**) or using either a sceptical or an enthusiastic prior (**c**)

In the whole modified ITT population, there was no evidence of any effect overall, as illustrated by the Bayesian posterior median HR of 60 day mortality estimated at 0.947 (95% credibility interval, 0.66–1.37), close to the frequentist estimate of 0.947 (95% confidence interval, 0.66–1.36) illustrating the non-informative priors (Fig. 1). Sensitivity analyses based on sceptical or enthusiastic prior only very slightly modified these findings (Fig. 1C). The posterior probability of a more than trivial benefit (HR < 0.95) and a more than trivial harm (HR > 1.05) in the DXM20 group was 0.51 and 0.29, respectively.

**Fig. 2:**
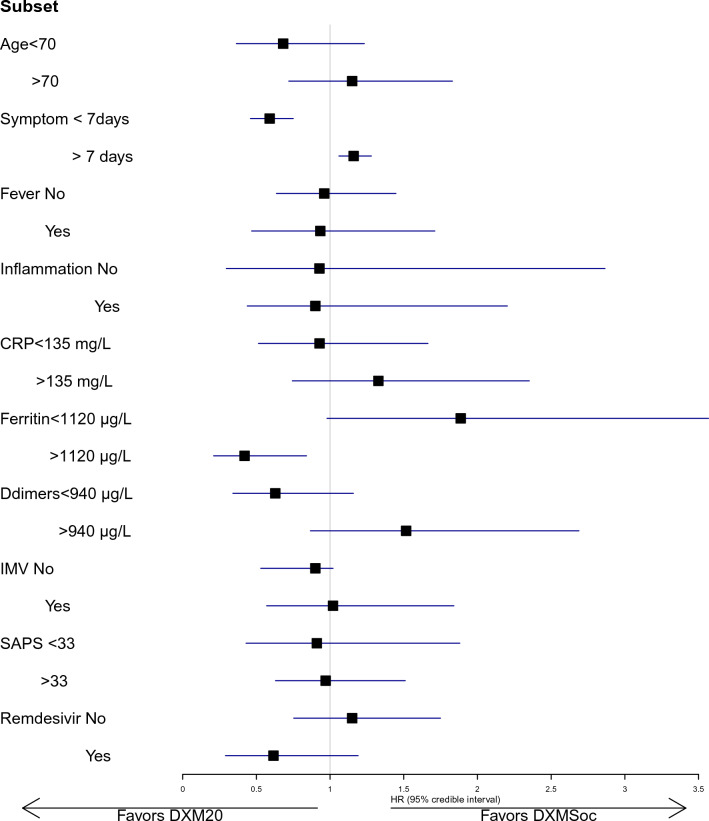
Looking for treatment by subset interactions in terms of hazard ratio (HR) of 60-day mortality. *CRP* C reactive protein, *MV* invasive mechanical ventilation, *SAPS* Simplified Acute Physiology Score

**Fig. 3:**
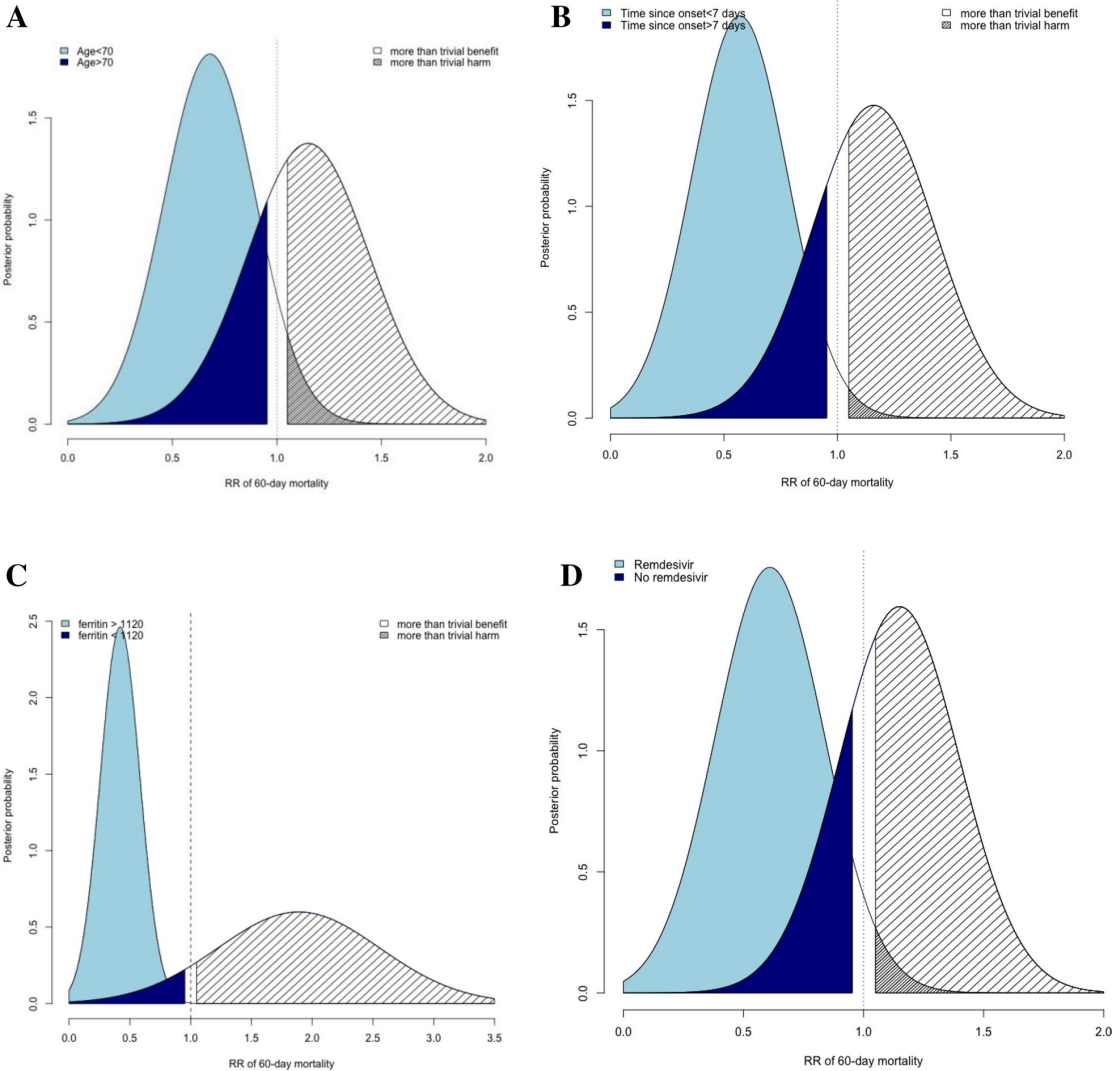
Posterior density of the hazard ratio of death within 60 deaths in DXM20 over DXMSoc group, according to subsets. Subsets were defined by age (< , > 70, Fig. 2a), by time since symptoms onset (< , > 7 days, Fig. 2b) and by remdesivir use or not (Fig. 2c)

Some evidence of a treatment-by-subset interaction, that is, heterogeneity of the treatment effect in some subsets, was suggested (Fig. 2, Table 2). First of all, this concerned the patient’s age: indeed, there was a 99.9% probability that the DXM20 benefit differed by at least 20%, with some evidence of benefit for patients aged under 70 years (HR = 0.68, 95% CrI 0.37–1.23, probability of benefit 86.5%, probability of harm 7.7%) while on the opposite some evidence of deleterious effect in those aged above 70 years (HR = 1.15, 95% CrI 0.71–1.83, probability of benefit 22%, probability of harm 64%) (Fig. 3A). Otherwise, high-dose DXM may have benefited patients with treatment onset within the first 7 days of infection (HR = 0.59, 95% CrI 0.46–0.75) while it was deleterious in those who were admitted later (HR = 1.16, 95% CI 1.06–1.28) (Fig. 3B), or in those with high levels of ferritin, with a 99% probability of benefit when ferritin > 1120 μg/L compared to 2.3% in those < 1120 μg/L (Fig. 3C, Table 2). Close findings were observed, though erased, according to CRP, with some evidence of decreased effect of DXM20 in patients with CRP > 135 (HR ratio of 1.43, 95% CrI 0.79–2.59). Similarly, patients with low levels of Ddimers appeared to have benefited from DXM20 (HR = 0.63, 95% CrI 0.34–1.17) compared to those with high levels in whom the treatment appeared deleterious (HR = 1.52, 95% CrI 0.87–2.72) (HR ratio = 2.41, 95% CrI 1.38–4.29). Finally, we observed an 91% probability of interaction between remdesivir use and DXM20 effect on Day 60 mortality, where remdesivir use was associated with a 90% chance of possible benefit of DXM20 (HR = 0.61, 95% CrI 0.29–1.18) compared to 19% in those who did not receive remdesivir (HR = 1.15, 95% CrI 0.75–1.75) (Fig. 3D). By contrast, there was no evidence of any treatment-by-subset interaction according to the fever (with a 0.58 posterior probability of HR differing by 20%) or SAPS2 (Probability of interaction of 0.67).

**Table 2:** Bayesian estimation of treatment effects of DXM20 vs DXMSoc across baseline subsets, looking for treatment-by-subset interactions

Subsets	Posterior quantities				
	HR death, 95% credible interval	Pr benefit (HR < 0.95)	Pr harm (HR > 1.05)	HR ratio (95% credible int)	Pr interaction (HR ratio > 1.2)
All	0.95 (0.66–1.37)	0.511	0.290		
Age, years					
< 70	0.68 (0.37–1.23)	0.865	0.077	1.00	0.84
$\ge$ 70	1.15 (0.72–1.83)	0.22	0.64	1.69 (0.79–3.66)	
Days since symptoms					
< 7	0.59 (0.46–0.75)	0.999	< 0.0001	1.00	0.99
$\ge$ 7	1.16 (1.06–1.28)	< 0.0001	0.981	1.99 (1.52–2.56)	
Body temperature, °C					0.58
< 38	0.96 (0.63–1.45)	0.487	0.335	1.00	
$\ge$ 38	0.93 (0.47–1.71)	0.526	0.357	0.97 (0.49–1.79)	
Inflammation^a^					
No	0.94 (0.30–2.79)	0.507	0.422	1.00	0.68
Yes	0.91 (0.44–2.16)	0.541	0.366	0.97 (0.44–2.59)	
CRP, mg/L					
< 135	0.93 (0.51–1.67)	0.533	0.340	1.00	0.76
$\ge$ 135	1.33 (0.74–2.38)	0.129	0.789	1.43 (0.79–2.59)	
Ferritin, μg/L					
< 1120	1.87 (0.96–3.57)	0.023	0.957	1.00	0.99
$\ge$ 1120	0.42 (0.21–0.84)	0.99	< 0.001	0.22 (0.09–0.58)	
Ddimers, ng/mL					
< 940	0.63 (0.34–1.17)	0.906	0.051	1.00	0.99
$\ge$ 940	1.52 (0.87–2.72)	0.050	0.904	2.41 (1.38–4.29)	
IMV					
No	0.90 (0.53–1.43)	0.600	0.254	1.00	0.65
Yes	1.02 (0.57–1.84)	0.401	0.463	1.14 (0.54–2.44)	
SAPS II					
< 33	0.91 (0.43–1.88)	0.55	0.35	1.00	0.67
$\ge$ 33	0.97 (0.63–1.51)	0.46	0.36	1.07 (0.46–2.57)	
Remdesivir use					
No	1.15 (0.75–1.75)	0.191	0.660	1.00	0.91
Yes	0.61 (0.29–1.18)	0.901	0.056	0.54 (0.25–1.03)	

In each subset, hazard ratio (HR) of death within the first 60 days (with 95% credibility intervals, CrI) were computed, with posterior probability of benefit or harm; then potential heterogeneity in effect across subsets was measured on the ratio of both HR

*CRP* C reactive protein, *O2* oxygen, *HFNO* High-Flow Nasal Oxygen, *CPAP* Continuous Positive Airway Pressure, *IMV* invasive mechanical ventilation, *IL6* interleukin 6, *SAPS* Simplified Acute Physiology Score, *HR* hazard ratio, *Pr* probability

^a^Defined as either ferritin level > 1000 μg/L or CRP > 100 mg/L

Sensitivity analyses are reported in Additional file. Modifying the prior in terms of baseline hazards or treatment effect did not affect the results (Additional file 1: Table S1). When ignoring time to death by modelling the prevalence rather than the hazard of death in the first 60 days, detected interactions were also roughly similar. Nevertheless, previous observed heterogeneity in treatment effect across D-dimers or ferritin levels were erased using a beta-binomial model, likely due to the fact that all survival curves reached close 60 day estimates (Additional file 1: Fig S2). By contrast, three main interactions, with age, time to symptoms onset, and remdesivir, were confirmed.

## Discussion

In the initial phase of the pandemic, large platform trials reported the benefit of corticosteroids (mainly low/intermediate doses) in SARS-CoV-2 AHRF [1, 2]. Recently, using all the available data included in a systematic review and meta-analysis [7], the Cochrane network concluded that systemic corticosteroids plus usual care probably reduces the number of deaths from any cause slightly, up to 30 days. No definite conclusion could be drawn about the number of deaths from any cause up to 120 days or on the optimal dose and duration of corticosteroids given to the patients.

In this post hoc analysis using a Bayesian approach, we first confirmed the absence of any treatment benefit on Day 60 mortality associated with high-dose DXM compared to low/intermediate doses of DXM (Table 2, Fig. 1). The Bayesian approach was considered, because it allows to reflect the uncertainty in treatment effect, illustrated through posterior densities of the HR of death. Moreover, by contrast to frequentist approaches, further probabilistic statements could be derived from these distributions, such as the probability of benefit or harm, as well as the probability that the HR of death differs by 20% from one subset to another. This allowed us to quantify a 51% posterior probability of a more than trivial benefit and a 29% of a more than trivial arm of high-dose DXM in the whole sample. This result contradicts the Bayesian secondary analysis of the COVID-STEROIDS2 study [23]. The adjusted RR for 28 day mortality was 0.87 (95% CI 0.73–1.03), with probabilities of any benefit, clinically important benefit, and clinically important harm of 94.8, 80.7, and 0.9%, respectively. In addition to the difference in outcomes, many patient characteristics were very similar, i.e., time from symptoms onset and randomization (9 days in the median in both studies), IMV rate (17 and 21%), and age (mean 65 vs. 67 years). However, one-third of the patients from COVID-STEROIDS 2 were enrolled in low-income countries, with one-fifth randomized outside of the ICU. Moreover, remdesivir was used more frequently in COVID-STEROIDS2 than in COVIDICUS (62 vs 26%).

The potential benefit of remdesivir in intensive care patients particularly those with IMV/ECMO patient remained a matter of uncertainty [35, 36]. A recent individual patient data meta-analysis showed that remdesivir reduced mortality in patients hospitalized with COVID-19 who required no or conventional oxygen support, but was underpowered to evaluate patients who were ventilated when receiving remdesivir. The effect size of remdesivir in patients with more respiratory support and the cost-effectiveness of remdesivir remain to be further elucidated [37]. On the opposite a cohort study based on the PREMIER database including more than 40,000 patients found a very significant benefit of the use of remdesivir in ECMO/IMV patients [38]. The importance of the viral load (maximal at the early phase of the disease) might be more important than the intensity of oxygenation deterioration in selecting patients accessible to remdesivir therapy.

The potential heterogeneity in the DXM20 effect according to the use of remdesivir observed in our study may explain some of the discrepancies between the effects of high-dose DXM in COVID-STEROIDS2 and COVIDICUS. Indeed, in our study, there was a 90% chance of high-dose DXM benefit in patients receiving remdesivir compared to less than 20% in those who did not (Table 2). This is in agreement with the reported effect of corticosteroid therapy in delaying viral clearance: a small effect towards delayed time to viral clearance in young treated patients compared to young untreated patients was found in a large epidemiologic study [39]. In the same study, viral dynamics after hospitalization was an independent predictor of mortality (HR = 1.31, *p* < 10–3). Finally, a secondary analysis of the DISCOVERY study comparing remdesivir with the standard of care found that remdesivir use was associated with a small but significant increase in viral clearance [40]. We can, therefore, postulate that the potential benefit of high-dose DXM in the inflammatory process is offset by a detrimental effect on viral clearance. Remdesivir therapy may suppress this deleterious effect.

We also found that the posterior HR of death in DXM20 vs DXMSoc was 0.59 when the time from symptoms onset was < 7 days, with an 99% probability of interaction between this delay and the DXM20 benefit; approximately one-quarter of both groups received remdesivir. In the RECOVERY study, a short delay between the first symptoms and randomization was associated with an insignificant impact of DXMSoc6 on Day 28 mortality suggesting that 6 mg dose was not large enough [1]. Time from symptoms onset to corticosteroid administration did not impact the corticosteroid effect in the Outcomerea cohort [16]. However, the interaction between time from symptoms onset and high dose benefit was not found in the COVID-STEROID2 trial [6]. One possible hypothesis is that inflammation is more important in patients whose respiratory status rapidly deteriorates with a possible higher benefit of a high dose of corticosteroids. This is in line with the 99% posterior probability of a beneficial effect in patients with high inflammation as reflected by a high ferritin level (above 1120 μg/L) compared to < 1% in those with lower levels. Unfortunately, the inflammation characteristics of the patients included in the COVID-STEROID2 study are not available.

The relationship between inflammatory reactions and corticosteroid effects was also suggested during the early phase of the pandemic, although based on observational data [27]. Using a latent class variable model, the authors found significant heterogeneity in the corticosteroid effect on mortality across inflammatory phenotypes, with corticosteroid exposure associated with decreased mortality in the hyperinflammatory phenotype and increased mortality in the hypoinflammatory phenotype [27]. Finally, an individualization of the corticosteroid dose based on the level of inflammation was suggested by a preliminary study but remains to be further evaluated [41]. The impact of hyperinflammation on the selection of patients who may benefit from high-dose DXM requires further study.

Our study has some limitations. We used a Bayesian modelling of the hazard of death. A multinormal model for the log hazard could have been used [30], but we choose to specify some model for the baseline hazard [42]. Thus, we used a piecewise exponential baseline hazard, with equally spaced knots while a random grid of timepoints could have been used [43]; nevertheless, the influence on posterior of prior specifications, including for the failure rates parameters, was evaluated and did not exhibit marked differences in results. The influence of inflammation status on the DXM20 effect differed according to the biomarker, and appeared more influenced by the ferritin level than by the CRP level, though discrepancies could rely on the choice of the cut-off points. We choose to rely on the literature or on the median value to limit overinterpretation of fishing. Moreover, the effect of Ddimers differed from that of ferritin; this could rely on sepsis-associated coagulopathy irrespective of the other inflammatory pathways [44]. Last, we focused our analyses of treatment by subset interaction to factors that have been evoked in previous studies of corticosteroid effects in SARS-CoV-2 ARF. Of course, other effect modifiers could have been considered. However, the use of Bayesian analyses to unmask possible effect modifiers is considered the best way to avoid enormous inflation of the risk of drawing erroneous conclusions [45]. Confirmed on all the sensitivity analyses, the potential interests of high dose dexamethasone when the delay from the first symptom is less than 7 days, in non-elderly patients or in combination with remdesivir ought to be further explored.

## Conclusions

Although no clear-cut evidence of an effect on 60-day mortality of high-dose corticosteroid therapy in patients with severe COVID-19 with ARF admitted to the ICU was observed, some subsets may benefit from such high-dose steroids. Heterogeneity in effects according to age, time to ICU admission, and concomitant use of remdesivir was evidenced. This might be emphasized by the concurrent use of remdesivir in prompting viral clearance. As previously this result remained to be confirmed but argue for the use of remdesivir when high dose of DXM is decided based on the short delay between the first symptoms and ICU admission. These hypotheses need to be confirmed in further studies.

### Supplementary Information

**Additional file 1: Table S1.** Treatment effect on 60-day mortality across subsets. **Figure S1.** Sensitivity analyses using either Cox model with sceptical or enthusiastic priors, or beta-binomial models with noninformative priors. **Figure S2.** Estimated survival curves according to DXM group and time since disease onset (A), D-dimers (B) or ferritin (C) levels

**Additional file 2.** Location and role of the collaborators.

## Data Availability

The datasets analyzed during the current study are not publicly available due to French regulation regarding trial data but are available from the corresponding author on reasonable request.

## Abbreviations

ARF: Acute respiratory failure
CrI: Credible interval
DXM: Dexamethasone
HR: Hazard ratio
ICU: Intensive care unit
RCT: Randomized clinical trial
RR: Relative risk
SARS-CoV-2: Severe acute respiratory syndrome coronavirus 2
