# Correction: Which severe COVID-19 patients could benefit from high dose dexamethasone? A Bayesian post-hoc reanalysis of the COVIDICUS randomized clinical trial

**DOI:** 10.1186/s13613-023-01194-x

**Published:** 2023-09-29

**Authors:** Sylvie Chevret, Lila Bouadma, Claire Dupuis, Charles Burdet, Jean‑Francois Timsit, the COVIDICUS RCT group

**Affiliations:** 1 ECSTRRA, UMR 1153, Saint Louis Hospital, University Paris Cité, Paris, France; 2 Medical and Infectious Diseases ICU, APHP Bichat Hospital, 75018 Paris, France; 3 Université Paris Cité, IAME, INSERM, UMR 1137, 75018 Paris, France; 4 Intensive Care Unit, Gabriel Montpied Hospital, CHU de Clermont-Ferrand, 63000 Clermont-Ferrand, France; 5 Epidemiology, Biostatistics and Clinical Research Department, AP-HP, Bichat Hospital, 75018 Paris, France

**Correction: Annals of Intensive Care (2023) 13:75** **https://doi.org/10.1186/s13613-023-01168-z**

In the original version of this article [1], the given and family names of the authors were swapped as Chevret Sylvie, Bouadma Lila, Dupuis Claire, Burdet Charles and Timsit Jean‑Francois. The corrected author names should read as Sylvie Chevret, Lila Bouadma, Claire Dupuis, Charles Burdet and Jean‑Francois Timsit. The original article has been corrected.
