# Supplementary material for: Which severe COVID-19 patients could benefit from high dose dexamethasone? A Bayesian post-hoc reanalysis of the COVIDICUS randomized clinical trial
Source: Ann Intensive Care. 2023 Aug 27;13:75. doi: 10.1186/s13613-023-01168-z (PMC10460760; doi:10.1186/s13613-023-01168-z)

**Table S1: Treatment effect on 60-day mortality across subsets**

The table reports the estimated effect on the risk of death within the first 60 days in the DXM20 group compared to the DXMSoc group, as measured by the relative risk (RR) through a beta-binomial model; analysis is based on Bayesian inference, which allows us to report probabilistic statements regarding the treatment effect. Benefit is measured on a RR<0.95, while harm is measured by a RR>1.05.

| Baseline subsets | DXMSoc,  No deaths/No pts | DXM20,  No deaths/No pts | RR of death, 95% Cred interval | Influence  Pr(benefit)/Pr(harm) | Ratio of RR, 95% Cred interval | Interaction  Pr (RR differ by 20%) |
| --- | --- | --- | --- | --- | --- | --- |
| **All** | **60/239** | **56/234** | **1.06 (0.77 to 1.44)** | **0.270/0.505** |  |  |
| Age, years  < 70  $\geq$ 70 | 27/144 (0.19)  33/95 (0.35) | 18/135 (0.13)  38/99 (0.38) | 0.74 (0.41-1.22)  1.12 (0.77-1.61) | 0.847/0.082  0.218/0.602 | 1.00  1.63 (0.81-3.01) | 0.801 |
| Days since symptoms onset  < 7  $\geq$ 7 | 22/55 (0.40)  38/183 (0.21) | 13/52 (0.25)  41/175 (0.23) | 0.659 (0.356-1.087)  1.163 (0.779-1.669) | 0.929/0.034  0.198/0.637 | 1.00  1.92 (0.93-3.61) | 0.895 |
| Body Temperature, °C  < 38  $\geq$ 38 | 46/186 (0.25)  13/49 (0.26) | 44/182 (0.24)  12/51 (0.23) | 0.99 (0.68-1.39)  0.95 (0.46-1.73) | 0.436/0.349  0.573/0.316 | 1.00  0.99 (0.43-1.93) | 0.637 |
| Inflammation syndrome*  No  Yes | 7/26 (0.27)  46/162 (0.28) | 6/23 (0.26)  36/151 (0.24) | 1.07 (0.386-2.353)  0.97 (0.682-1.337) | 0.475/0.438  0.747/0.120 | 1.00  1.11 (0.38-2.61) | 0.703 |
| CRP, mg/L  < 135  $\geq$ 135  Missing | 24/98 (0.24)  27/99 (0.27)  9/42 (0.21) | 22/98 (0.22)  23/98 (0.23)  11/38 (0.29) | 0.95 (0.558-1.515)  0.89 (0.534-1.389) | 0.558/0.298  0.657/0.207 | 1.00  1.01 (0.47-1.89) | 0.603 |
| Ferritin, mg/L  < 1120  $\geq$ 1120  Missing | 21/76 (0.28)  17/61 (0.28)  22/102 (0.22) | 17/61 (0.28)  17/77 (0.22)  22/96 (0.23) | 1.047 (0.587-1.718)  0.832 (0.450-1.423) | 0.410/0.444  0.731/0.171 | 1.00  0.86 (0.36-1.74) | 0.697 |
| D-Dimers, ng/mL  < 940  $\geq$ 940  Missing | 21/89 (0.24)  31/103 (0.30)  8/47 (0.17) | 21/103 (0.20)  29/88 (0.33)  6/43 (0.14) | 0.898 (0.512-1.459)  1.120 (0.720-1.663) | 0.638/0.238  0.259/0.577 | 1.00  1.34 (0.65-2.48) | 0.672 |
| IMV  No  Yes | 37/143 (25.9)  23/96 (24.0) | 32/137 (23.4)  24/97 (24.7) | 0.925 (0.599-1.359)  1.062 (0.633-1.673) | 0.595/0.237  0.376/0.468 | 1.00  1.20 (0.60-2.14) | 0.599 |
| SAPS II  <33  $\geq$ 33 | 16/113 (14.2)  41/109 (37.6) | 13/100 (13.0)  41/110 (37.3) | 0.979 (0.477- 1.788)  1.007 (0.704-1.397) | 0.532/0.353  0.406/0.371 | 1.00  1.15 (0.51-2.26) | 0.634 |
| Remdesivir use  No  Yes | 43/178 (0.24)  17/61 (0.28) | 46/172 (0.27)  10/62 (0.16) | 1.124 (0.776-1.583)  0.625 (0.294-1.137) | 0.206/0.609  0.923/0.043 | 1.00  0.58 (0.24-1.14) | 0.892 |

* defined as either ferritin level > 1,000 mg/L or CRP > 100 mg/L

**Figure S1: Sensitivity analyses using either Cox model with sceptical or enthusiastic priors, or beta-binomial models with noninformative priors**


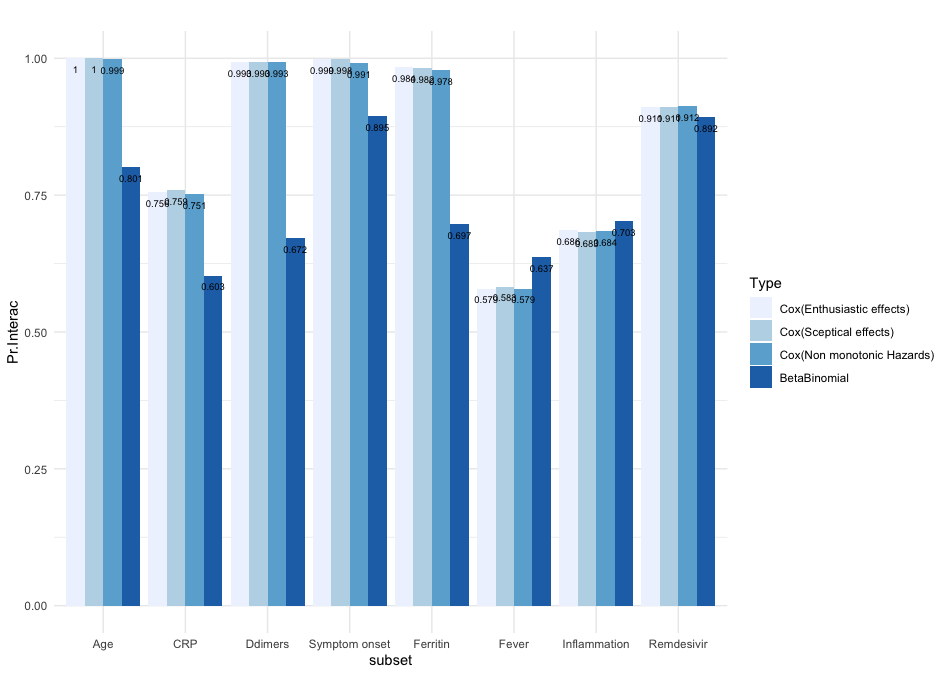


**Figure S2: Estimated survival curves according to DXM group and time since disease onset (A), D-dimers (B) or ferritin (C) levels**

Fig S2A Fig S2B


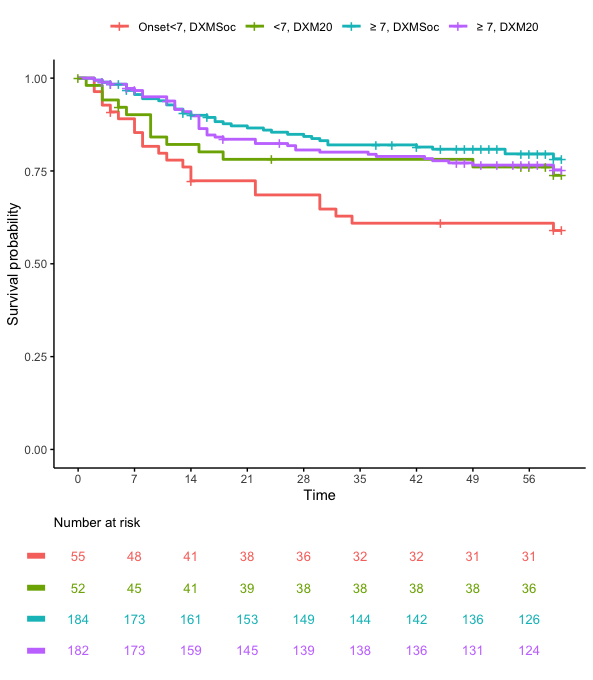

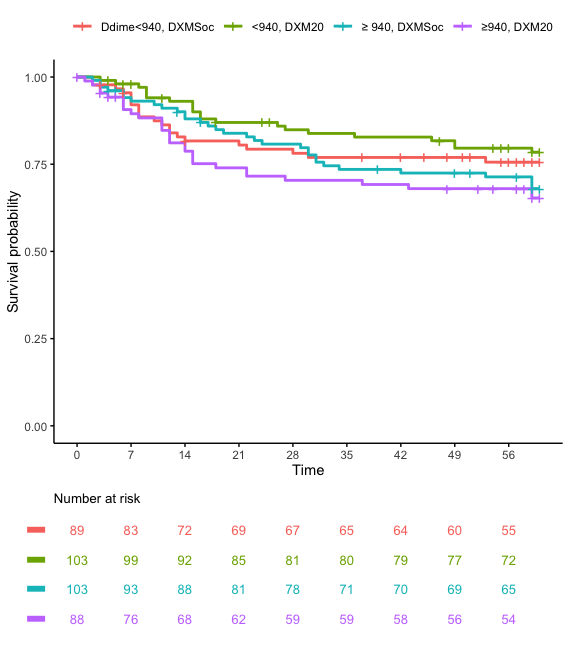


Fig S2C


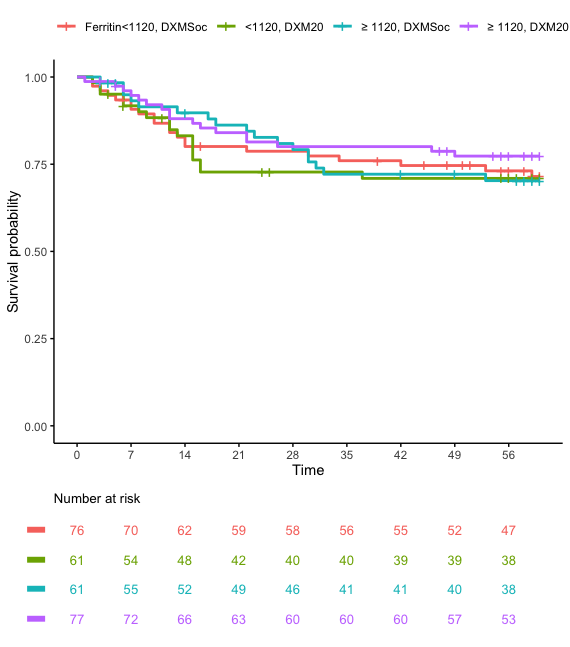

Supplement: Supplementary file 1 — Additional file 1: Table S1. Treatment effect on 60-day mortality across subsets. Figure S1. Sensitivity analyses using either Cox model with sceptical or enthusiastic priors, or beta-binomial models with noninformative priors. Figure S2. Estimated survival curves according to DXM group and time since disease onset (A), D-dimers (B) or ferritin (C) levels [file 13613_2023_1168_MOESM1_ESM.docx]
